# First Principles Theory of the hcp-fcc Phase Transition in Cobalt

**DOI:** 10.1038/s41598-017-03877-5

**Published:** 2017-06-19

**Authors:** Raquel Lizárraga, Fan Pan, Lars Bergqvist, Erik Holmström, Zsolt Gercsi, Levente Vitos

**Affiliations:** 1 Applied Materials Physics, Department of Materials Science and Engineering, Royal Institute of Technology (KTH), Stockholm, SE-10044 Sweden; 2 Department of Materials and Nano Physics, School of Information and Communication Technology, Royal Institute of Technology (KTH), Electrum 229, SE-16440 Kista, Sweden; 3 Swedish e-Science Research center (SeRC), Royal Institute of Technology (KTH), SE-10044 Stockholm, Sweden; 4 Sandvik Coromant R&D, Stockholm, SE-12680 Sweden; 5 School of Physics and CRANN, Trinity College, Dublin 2, Ireland; 6 Department of Physics and Astronomy, Division of Materials Theory, Uppsala University, Box 516, SE-75121 Uppsala, Sweden; 7 Research Institute for Solid State Physics and Optics, Wigner Research Center for Physics, P.O. Box 49, Budapest, H-1525 Hungary

## Abstract

Identifying the forces that drive a phase transition is always challenging. The hcp-fcc phase transition that occurs in cobalt at ~700 K has not yet been fully understood, although early theoretical studies have suggested that magnetism plays a main role in the stabilization of the fcc phase at high temperatures. Here, we perform a first principles study of the free energies of these two phases, which we break into contributions arising from the vibration of the lattice, electronic and magnetic systems and volume expansion. Our analysis of the energy of the phases shows that magnetic effects alone cannot drive the fcc-hcp transition in Co and that the largest contribution to the stabilization of the fcc phase comes from the vibration of the ionic lattice. By including all the contributions to the free energy considered here we obtain a theoretical transition temperature of 825 K.

## Introduction

Phase transitions are one of the most fundamental phenomena of matter. Understanding the driving forces behind them enables development of new theories, discoveries and tailor-design of new materials. Pressure-induced phase transitions are routinely investigated by means of density functional theory (DFT) based methods^1^. However, it is still a great challenge to describe the mechanisms and forces that control phase transformations induced by a change in temperature.

The temperature-induced phase transition in Co is unique among the elements of the periodic table^2^. Right below 700 K, Co undergoes a phase transition from the low temperature hexagonal close-packed (hcp) phase to the high temperature face-centered cubic (fcc) phase^3^. Although, both fcc and hcp phases, are present in the temperature-pressure phase diagram of many elements, for example, rare earths and heavy actinides, only direct transitions between these two phases occur in He, Fe, Co, Tl, Pb and Yb. Moreover, among these few elements, Co is the only one that does not have the body-centered cubic (bcc) phase as well in its phase diagram. This means that standard mechanisms such as the Bain deformation can not be used to describe the phase transition in Co^4^.

**Figure 1:**
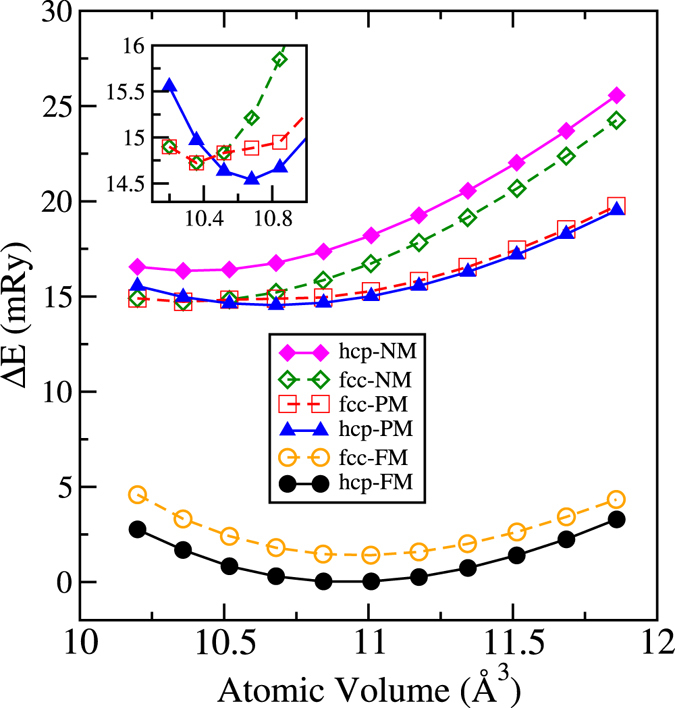
Calculated total energies for Co in the hcp and fcc phases in three magnetic states; FM (full- and open-circles, respectively), NM (full- and open diamonds, respectively) and PM (full-triangles and open-squares, respectively). Energies are given with respect to the energy of the FM hcp structure at the equilibrium volume. The inset shows a zoom-in for the fcc-NM, fcc-PM and hcp-PM curves near their minima.

Magnetism plays an important role in the phase stability of the 3*d* transition metals^5–8^. As a matter of fact, the 3*d* magnetic elements do not follow the crystal structure sequence, hcp → bcc → hcp → fcc from left to right in the periodic table, found in the non-magnetic transition metals. Skriver was able to explain this sequence using a *d*-band filling argument^9^. Later on, Söderlind *et al*. extended Skriver’s theory to account for the magnetic 3*d* elements using the fractional filling of both, spin-up and spin-down sub-bands^10^. Following these arguments one can conclude that if Co was not magnetic it would choose the fcc phase as a ground state. Indeed, density functional theory (T = 0 K), shows correctly that the ferromagnetic (FM) hcp is the ground state (Fig. 1) but if magnetism is ignored the non-magnetic (NM) fcc phase is lower in energy than the NM hcp.

The general consensus has been so far that the hcp ground state in Co is stabilized by magnetism and that at higher temperatures, the reduction of magnetism destabilizes the hcp phase, and thereby restoring the natural tendency of Co to be fcc^8, 10, 11^. Two decades ago, Uhl and Kübler concluded from their theory that spin-fluctuations and reduced magnetization at higher temperatures lower the free energy of the fcc phase with respect to the hcp, triggering the phase transition^8, 12^. They reported a calculated transition temperature *T* = 590 K from magnetic effects only, however, they did not rule out other mechanisms such as phonons, as responsible of the structural transformation.

Because the structural transition in Co takes place deep inside the ferromagnetic regime, a much more complex scenario is anticipated. As opposed to the case of Fe, where the bcc-fcc structural transformation occurs 140 K above the Curie temperature, the fcc-hcp transition in Co takes place far below the Curie temperature ~1400 K^11^. Furthermore, the magnitude of the local magnetic moments is reduced only about 8% at the Curie temperature^13^ which suggests that at the structural transformation the local moments are not significantly altered.

In this work, we show from a comprehensive analysis of the free energy with first principles theory, that the weakening of magnetism alone is not sufficient to destabilize the hcp phase of Co at 700 K. Our study reveals that vibrational energy constitutes the main driving force behind the hcp-fcc structural transition and that all the free energy terms studied here, that is, vibrational, magnetic, electronic and volume effects, must be included to understand it properly.

## Results and Discussion

Over the past thirty years, the magnetic, electronic and structural properties of Co have been extensively investigated by means of density functional theory (DFT) calculations^5, 6, 11, 16–21^. In Fig. 1 we present exact muffin-tin orbitals (EMTO)^22^ total energy calculations for different magnetic configurations: FM, NM and paramagnetic (PM). The PM was modeled by using the disordered local moments approach (DLM)^23, 24^. In the figure, energies are given with respect to the total energy of the FM hcp structure at the equilibrium volume, *V*_0_ = 10.93 Å^3^. Our results agree well with experiments *V*_exp_ = 11.07 Å^3^ ^3^ and previous calculations, correctly describing the FM hcp as the ground state^5, 25^. The equilibrium volumes for PM calculations are slightly lower than the FM one; 10.65 Å^3^ for hcp and 10.39 Å^3^ for fcc. For small volumes (≤10.56 Å^3^) the PM fcc curve coincides with the NM one because PM fcc looses the local magnetic moments. What is interesting to note is that the PM curves for both, hcp and fcc, are almost degenerate. This is quite remarkable because, contrary to what one expects, the fcc phase in the PM state is not lower in energy than the PM hcp. Since we believe that the PM state at higher temperatures is well described by DLM, one must conclude that the withdrawal of the FM ordering does not destabilize hcp and therefore other mechanisms must be invoked to explain the structural transition.

**Figure 2:**
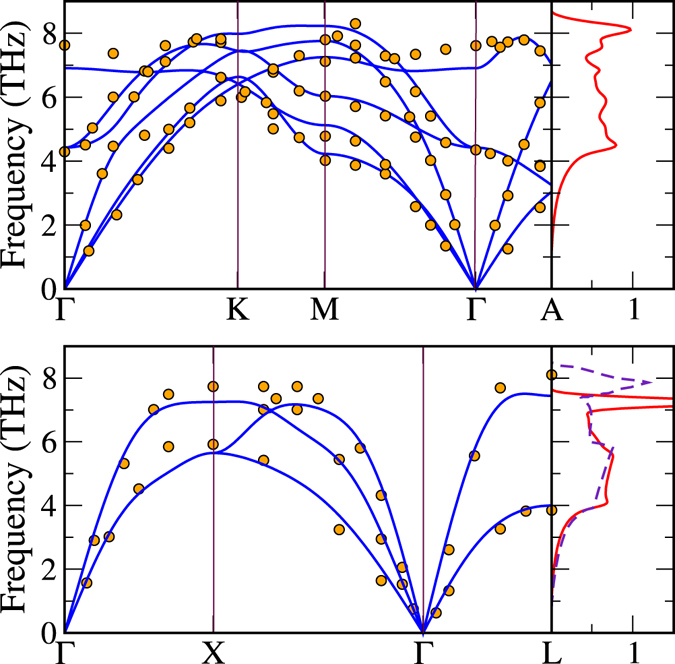
In the top-left panel the phonon dispersion relation along high symmetry lines for FM hcp Co is shown. The experimental values (dots) are taken from ref. 14. The calculated hcp phonon DOS is shown to the right of the figure. In the bottom-left panel the phonon dispersion relation along some high symmetry lines for FM fcc Co is displayed. The experimental values were obtained for Co_0.92_Fe_0.08_ (dots) in ref. 15. The fcc phonon DOS and the calculated DOS obtained from the phonon spectra^15^ (dashed-line) for fcc Co_0.92_Fe_0.08_ are presented to the right of the figure.

In order to study the contribution of vibrational energy to the free energy we performed phonon calculations within the density functional perturbation theory^26^. The phonopy software^27, 28^ and the Vienna ab initio Simulation Package^29^ (VASP) were used to calculate force constants. Details of the calculations were chosen in accordance to EMTO calculations. In Fig. 2 we show the phonon density of states (DOS) of FM hcp and FM fcc. The phonon spectra of pure fcc Co has not been measured because the structural transformation at 700 K is usually destructive, however several lattice dynamics studies exist for Co_0.92_Fe_0.08_^15, 30^. The authors in those studies argued that due to the fact that Fe and Co have similar atomic masses and sizes, Co_0.92_Fe_0.08_ should be representative of pure fcc Co. Shapiro *et al*. used the peak positions of the phonon spectra of fcc Co_0.92_Fe_0.08_ to calculate a phonon DOS^15^, shown (dashed line) in Fig. 2 for comparison. The phonon calculations were performed using theoretical volumes at *T* = 0 K. Our fcc phonon DOS agrees well with the experimental DOS concerning the shape, however, the width of the DOS is underestimated by ~8% and the peak position at around 7.8 THz is 0.5 THz lower in the calculations. It is interesting to note that the fcc phonon DOS grows faster than the hcp phonon DOS up to 4 THz, which implies that the fcc phase gets access to more entropy than hcp before 4 THz and therefore fcc lowers more its free energy, promoting the phase transformation.

In Fig. 2 we show also phonon dispersion relations for Co along selected high symmetry lines for FM hcp (top panel) and FM fcc (bottom panel). Dots in both panels correspond to experiments in refs 14 and 15, respectively. The phonon spectrum for hcp Co is very well described by our phonon calculations, and only small discrepancies appear at higher frequencies around 7 THz along the ΓM direction and the ΓK direction. Our results are in excellent agreement with other DFT phonon calculations for the hcp phase^31^. The fcc phonon spectrum also compares very well with experiments. At higher frequencies, we find that the maximum frequencies along the three directions are ~0.5 THz lower than experiments. These discrepancies may be due to the presence of Fe in the fcc Co_0.92_Fe_0.08_ phonon spectrum.

In order to discuss the stability of the two phases, we apply the equilibrium condition, Δ*F* = 0, where the free energy difference can be expressed as^32^

1 $${\rm{\Delta }}F={\rm{\Delta }}{U}_{{\rm{st}}}+{\rm{\Delta }}{F}_{{\rm{vib}}}+{\rm{\Delta }}{F}_{{\rm{el}}}+{\rm{\Delta }}{F}_{{\rm{m}}}+{\rm{\Delta }}{F}_{{\rm{anh}}}+{\rm{\Delta }}{F}_{{\rm{ep}}}\mathrm{.}$$

**Figure 3:**
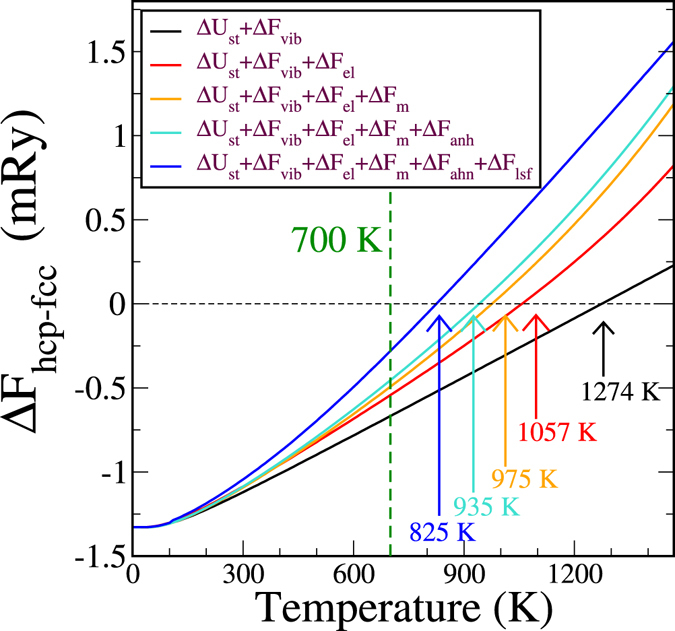
Temperature dependence of the free energy differences. The solid black curve contains the contribution from the static energy and the phonon free energy, the red one adds the electronic contribution, the orange one adds the magnetic free energy, the cyan one contains as well a quasi-harmonic contribution and the blue one contains an estimation of the LSF contribution.

The six terms in Eq. 1 correspond to the static energy ΔU_st_ as obtained by DFT calculations at T = 0 K, the vibrational free energy due to phonons Δ*F*_vib_, electronic excitations Δ*F*_el_, magnetic free energy Δ*F*_m_, the anharmonic term which accounts for volume expansion Δ*F*_anh_, and the electron-phonon interaction Δ*F*_ep_. These terms have been added successively in Fig. 3 to show the effect of each one of them, except the last one, Δ*F*_ep_, that is likely to be relatively small as we will discuss later. The black solid curve in Fig. 3 corresponds to the static energy plus the phonon contribution. It crosses the zero-axis at 1274 K which is higher than the observed transition temperature. This shows that vibrations of the ionic lattice can stabilize fcc against hcp by themselves, however other contributions to the free energy are expected to lower the transition temperature further.

**Figure 4:**
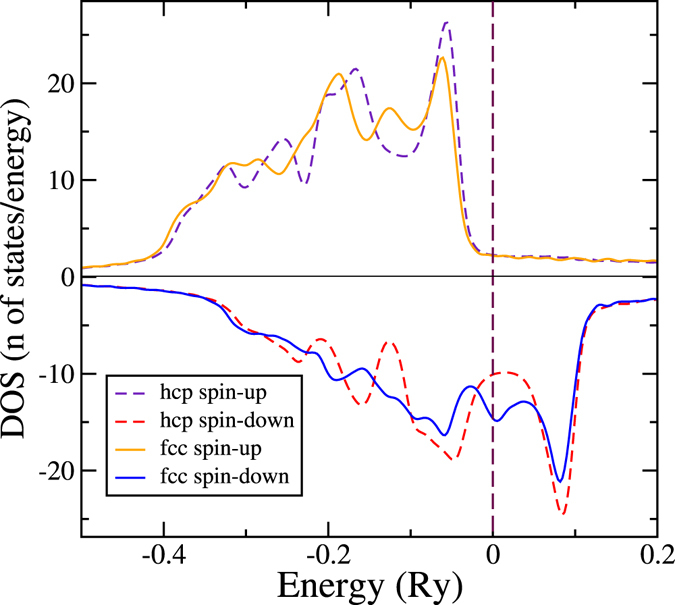
Spin projected density of states for the Co hcp and fcc phases. The Fermi level is indicated by a dashed vertical line at 0 Ry.

The next term in Eq. 1 is the effect of electronic excitations and it has been added to the two previous terms (the red solid curve in Fig. 3). It can be seen that Δ*F*_el_ lowers the transition temperature to 1057 K. It is not obvious that this term should have such a strong influence. The origin of this can be found in the electronic DOS, since the electronic entropy, at least to a first approximation, is proportional to the electronic DOS at the Fermi level^33^. Figure 4 displays the spin projected DOS of FM Co in the hcp and fcc phases. The spin up DOS, for both hcp and fcc, are below the Fermi level and the *d*-states are completely occupied, whereas the spin down DOS cuts through the Fermi level. One can see that the fcc spin-down DOS has a peak at the Fermi level while the hcp has a valley. This difference accounts for a substantial contribution of the electronic entropy to decrease the free energy of fcc and hence the large reduction of the transition temperature. The features of the spin-up and spin-down DOS are consistent with other DFT calculations^34^. This unusually large contribution of the electronic excitations has been encountered before, for example, in Fe-Mg alloys at Earth’s inner core conditions. In that work a similar effect is calculated for bcc and hcp Fe and explains why bcc Fe becomes dynamically unstable at core conditions^35^. Additionally, we investigated the electron-phonon interaction effects in the electronic entropy by following the analysis of Grimvall^33^ and concluded that the effective contribution to the free energy is negligible in the case of cobalt.

The next contribution to the free energy in Eq. 1 comes from the magnetic system. In order to obtain the finite-temperature magnetic properties of Co beyond the DLM estimate in Fig. 1 we performed standard Monte Carlo calculations of a Heisenberg Hamiltonian with fixed-size magnetic moments by means of the Metropolis algorithm^36^. The calculated Curie temperatures are 1249 K for hcp and 1228 K for fcc Co. The contribution of the magnetic free energy can be seen in Fig. 3 (orange solid line). Our results show that the magnetic free energy pushes the transition temperature down to 975 K. We note here that the calculated magnetic free energy contribution itself does not stabilize fcc phase before the Curie temperature is reached.

Next we account for volume expansion effects which have been treated in the quasi-harmonic approximation as follows. The Gibbs free energy of a solid can be expressed as $G(T)={U}_{{\rm{st}}}+{E}_{{\rm{vib}}}(T,V)+PV\mathrm{.}$ Within the quasi harmonic approximation^27, 28, 37^,

2 $$G(T,P)={{\rm{Min}}}_{V}[{U}_{{\rm{st}}}+{E}_{{\rm{vib}}}(T,V)+PV]\mathrm{.}$$

The solid cyan curve in Fig. 3 contains the anharmonic contribution obtained from the Gibbs free energy in Eq. 2. The anharmonic term brings down the transition temperature to 935 K and constitutes the smallest contribution of all, which is not unexpected since the volume change during the transition Δ*V*/*V* = 3.3 × 10^−3^ is very small^4^.

**Figure 5:**
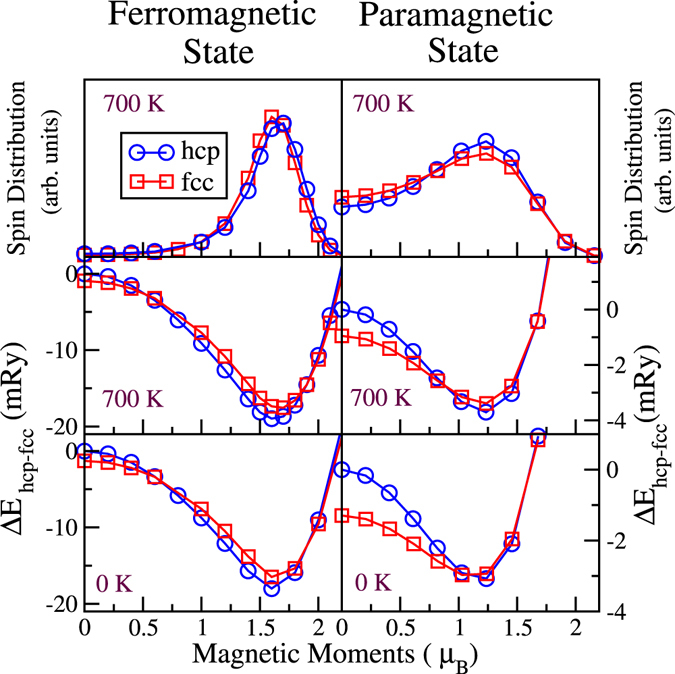
Results for the FM state and PM state are displayed in the left- and right-panels, respectively. In the top panel we show the spin density distribution for both hcp and fcc at 700 K. The bottom and middle panels show the fixed-spin total energies with respect to the hcp NM state, calculated for hcp and fcc at volumes corresponding to 0 K and 700 K, respectively.

The calculated transition temperature, 935 K, is higher than the observed temperature, which indicates that there are overlooked contributions to the free energy. Our Monte Carlo simulations contain the effect of transversal spin fluctuations but they do not include longitudinal ones. In order to estimate the contribution of longitudinal spin fluctuations (LSF) to the free energy we followed the methodology by Dong *et al*.^24^. We performed fixed-spin calculations^6^ to obtain a spin density distribution (SDD) corresponding to a temperature *T*,

3 $${p}_{i}=(\frac{1}{Z})\exp [-\frac{{E}_{i}}{{k}_{B}T}],$$

where *E*_*i*_ is the energy of a given magnetic state with a local magnetic moment *μ*_*i*_ and *Z* is the partition function. Fixed-spin total energies were calculated at volumes corresponding to 0 K and 700 K (V_hcp_ = 11.12 Å^3^ and V_fcc_ = 11.19 Å^3^) according to Eq. 2 and are displayed in the bottom and middle panels in Fig. 5. This figure contains results for the FM (left-panels) and the PM (right-panels) states. The PM results are included here for comparison only. We produced continuous SDD *P*(*μ*) by interpolation of {*E*_*i*_} (bottom and middle panels) and used them to calculate the LSF entropy and internal energy by means of the integral expressions

4 $$S=-{k}_{B}\,\int {\mathscr{P}}\,ln({\mathscr{P}})\,d\mu \,{\rm{a}}{\rm{n}}{\rm{d}}\,E=\int {\mathscr{P}}\,E(\mu )\,d\mu .$$

Finally we obtain ${\rm{\Delta }}{F}_{lsf}={\rm{\Delta }}E-T{\rm{\Delta }}S$ from the FM SDD and add this contribution to the free energy in Fig. 3 (blue solid line). LSF obtained from FM SDD decreased the transition temperature to 825 K.

Here, we would like to discuss briefly the results obtained by using the PM SDD (top-right panel). First we observe that the energy calculations for fcc are more sensitive to the volume than the ones for hcp. At T = 0 K, the energy curve for fcc has a shallower minimum which makes excitations energetically more favorable for fcc than hcp. This effect is not so pronounced at 700 K. By comparing the plots for T = 0 K and T = 700 K one can realize that not taking into account the volume expansion, that is, using the volume at zero temperature would result in an overestimation of the effect of LSF in the stabilization of fcc. This can also be seen in the FM case, but it is less obvious because the minima are much deeper. Therefore, it is important to consider volume expansion effects to correctly describe the influence of LSF.

## Conclusions

Up until now, the dominating energy contribution behind the structural fcc-hcp phase transition in Co has not been completely elucidated. Spin fluctuations and the reduction of the magnetic moments were discussed earlier to be responsible of the phase transition^8^. This early explanation is at odds with the fact that fcc Co is still magnetic up to ~1400 K and that the magnitude of the local magnetic moments are reduced around 8% at the Curie temperature^13^. Furthermore, the DLM calculations presented here also show that the disorder of the magnetic moments is not enough to lower the energy of the fcc phase with respect to the hcp phase.

In fact, the hcp phase is the ground state of Co and the energy difference between hcp and fcc is about 1.5 mRy at T = 0 K. We have demonstrated that as the temperature increases all temperature-dependent energy contributions included in this study favor fcc because all of them bring the energy of the fcc phase closer to that of the hcp phase. Finally, at the calculated transition temperature, 825 K, the energy gap of 1.5 mRy is closed up and consequently fcc becomes stable. However, the collective impact of all the energy contributions evaluated here, that is, the energy of the vibrations of the lattice, magnetic and electronic entropies and the volume expansion effect, need to be present otherwise fcc will be stabilized at a much higher temperature than observed. Therefore, from our analysis of the relevant components of the free energy of both phases we concluded that the magnetic effect alone can not stabilize^38^ the fcc phase at 700 K. We have also shown that vibrations of the ionic lattice are the largest energy contribution to the destabilization of hcp. Understanding the hcp-fcc phase transition in pure Co is very important for the study of the structural transition in Co-based alloys and compounds, that are currently being extensively used for industrial applications.

## Methods

Total energies were computed by two first principles methods; the exact muffin-tin orbitals (EMTO)^22^ and the Vienna ab-initio Simulation Package (VASP)^29, 39^. Both methods were tested carefully. At the equilibrium volume, the total energy difference between both phases was ~1.5 mRy in both methods, which is also in perfect agreement with other DFT methods^8^.

### EMTO

The Perdew, Burke, and Ernzerhof (PBE) parametrization of the exchange-correlation functional was used^40^. The paramagnetic state was modeled by the disordered local moments (DLM) approach in combination with the coherent potential approximation (CPA)^41^, in which Co is viewed as a 50–50 alloy, Co ${{\rm{Co}}}_{50}^{\uparrow }$ ${{\rm{Co}}}_{50}^{\downarrow }$ with randomly distributed magnetic moments^42^.

Calculations were performed with 637 and 505 special k-points in the irreducible Brillouin zone for the hcp and fcc phase, respectively. These number of k-points were chosen to minimize the convergence error for each phase within 10 *μ*Ry.

### VASP

We performed phonon calculations within the framework of density functional perturbation theory^26^ as implemented in VASP, that is based in the the projector augmented waves approach^43^ (PAW). In accordance with the EMTO calculations we used the PBE parametrization of the exchange-correlation functional^40^. The software Phonopy^27, 28^, that is an open source package for phonon calculations at harmonic and quasi-harmonic levels, was employed to determine phonon dispersion relations, the phonon density of states and the vibrational free energy. Supercells with 125 atoms for the hcp phase and 250 atoms for the fcc phase were used to obtain converged force constants.

Internal convergence parameters were carefully checked so that energy differences were below 10^−8^ eV per cell and an energy cutoff of 500 eV was used.

### Monte Carlo

In order to obtain the finite-temperature magnetic properties of Co we performed standard Monte Carlo calculations of a Heisenberg Hamiltonian with fixed-size magnetic moments by means of the Metropolis algorithm^36^.

We applied the magnetic force theorem^44^ and the Liechtenstein-Katsnelson-Antropov-Gubanov (LKAG) formalism^45, 46^ as implemented in the self-consistent field multiple-scattering Korringa-Kohn-Rostoker (KKR) Green’s function approach^47, 48^ to calculate the exchange coupling constants. The FM state was used as a reference state. In our Monte Carlo calculations we employed exchange constants of up to 5 nearest neighbors. The calculated Curie temperature was 1249 K for hcp and 1228 K for fcc Co.
